# Isolation of 3-amino-4-nitro­benzyl acetate: evidence of an undisclosed impurity in 5-amino-2-nitro­benzoic acid

**DOI:** 10.1107/S2056989015008750

**Published:** 2015-05-13

**Authors:** Brandon Quillian, Jordan Hendricks, Matthew Trivitayakhun, Clifford W. Padgett

**Affiliations:** a Department of Chemistry and Physics, Armstrong State University, 11935 Abercorn Street, Savannah GA 31419, USA

**Keywords:** crystal structure, 3-amino-4-nitro­benzyl acetate, intra­molecular, inter­molecular, resonance-assisted hydrogen bonding, 5-amino-2-nitro­benzoic acid

## Abstract

Yellow crystals of the title compound 3-amino-4-nitro­benzyl acetate, C_9_H_10_N_2_O_4_, were isolated from the reaction of acetic anhydride with (5-amino-2-nitro­phen­yl)methanol, prepared from reduction of commerically available 5-amino-2-nitro­benzoic acid with borane–THF. The mol­ecule is essentially planar (r.m.s. deviation = 0.028 Å). The mol­ecules are linked by inter­molecular N—H⋯O hydrogen-bonding inter­actions between the carbonyl and amine groups, forming a zigzag chain along the *b*-axis direction lying in a plane parallel to (-102). The chains are stacked along the *c* axis by π–π inter­actions [centroid–centroid distances = 3.6240 (3) and 3.5855 (4) Å]. A strong intra­molecular N—H⋯O hydrogen-bonding inter­action is observed between the nitro group and the amine group [2.660 (2) Å].

## Abstract 2

The crystal stucture of 3-amino-4-nitro­benzyl displays intra­molecular resonance-assisted hydrogen bonding between the *ortho* amino and nitro groups in addition to an inter­molecular network of hydrogen bonding and π-stacking.

## Chemical Context   

Often commercially available chemicals are sold with minor impurities in the range 1–5%; the user may choose to ‘use as received’ or further purify. The identities of the impurities are rarely disclosed in fine chemicals. Though these impurities may serve as benign spectators, in some cases they might hinder reactivity and/or produce undesirable by-products that are difficult to separate from the desired product. Therefore, it is important to identify these impurities to allow the users to decide if further purification is warranted. We recently purchased 5-amino-2-nitro­benzoic acid from Acros Organics^©^ (5 g, 97%, AC33074-0050) for our ongoing studies of photo-induced deca­rboxylation of *ortho*-nitro­benzyl esters (Cabane *et al.*, 2010 ▸; Pocker *et al.*, 1978 ▸). The isolation of the title compound, 3-amino-4-nitro­benzyl acetate, after the reaction of crude (5-amino-2-nitro­phen­yl)methanol, prepared from the reduction of 5-amino-2-nitro­benzoic acid, with acetic anhydride suggests 3-amino-4-nitro­benzoic acid is an impurity in the commercially available starting material.
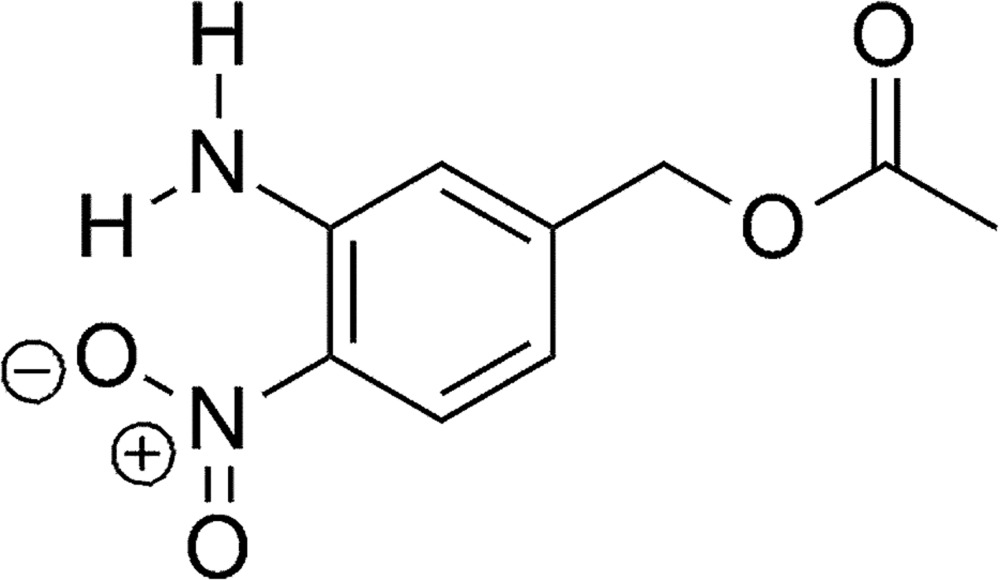


## Structural Commentary   

**Figure 1:**
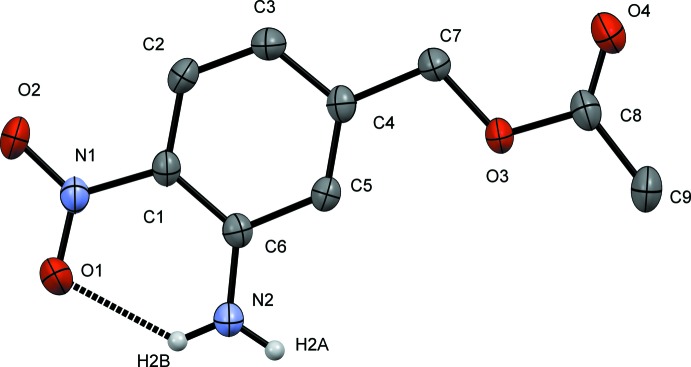
A displacement ellipsoid plot of 3-amino-4-nitro­benzyl acetate (50% probability level). C-bound H atoms have been omitted for clarity.

The asymmetric unit of the title compound (Fig. 1 ▸) displays an essentially planar mol­ecule (r.m.s.d. 0.028 Å) with the amine, nitro and acetate groups resting in the plane of the arene. The carbonyl, C=O [1.208 (2) Å], and ester, C—O [1.3477 (19) Å], bond distances are unassuming. The nitro bond distances [O1—N1 1.2500 (16) and O2—N1 1.2401 (17) Å] are similar to those in *N*-(3-chloro­phen­yl)-3-nitro­pyridin-2-amine [1.222 (2) and 1.245 (2) Å] (Aznan *et al.*, 2011 ▸). Atom O1 of the nitro group is involved in strong intra­molecular hydrogen bonding [graph set *S*1, 1(6)] between H2*B* of the amine at a distance of 2.06 (2) Å, forming a rigid, thermodynamically stable six-membered ring (Fig. 1 ▸). The elongated O1—N1 bond distance, as compared to the O2—N1 distance, is consistent with resonance-assisted hydrogen bonding between O1 and H2*B* (Beck & Mo, 2006 ▸).

## Supra­molecular Features   

**Figure 2:**
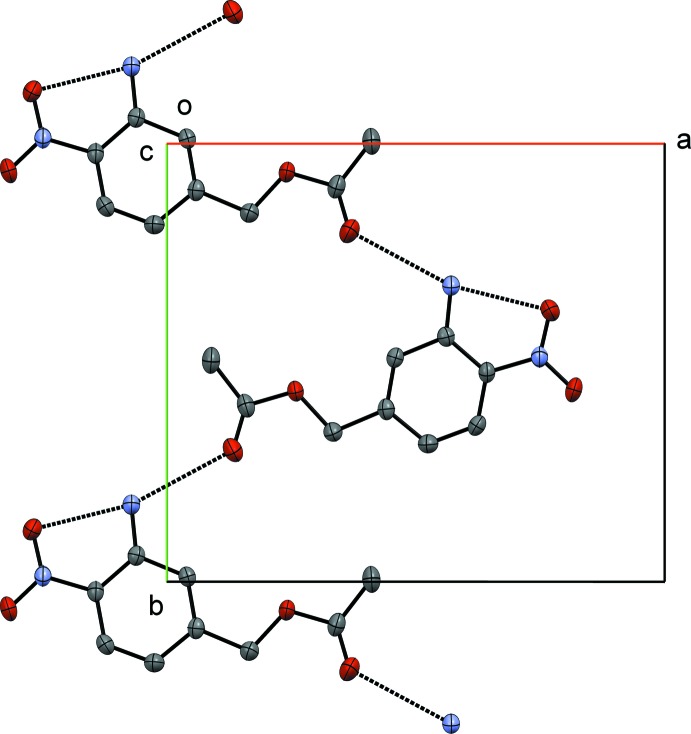
A single of layer of the unit cell of 3-amino-4-nitro­benzoic acid through the *ab* plane (observed down the *c* axis), highlighting the hydrogen-bonding motif.

**Figure 3:**
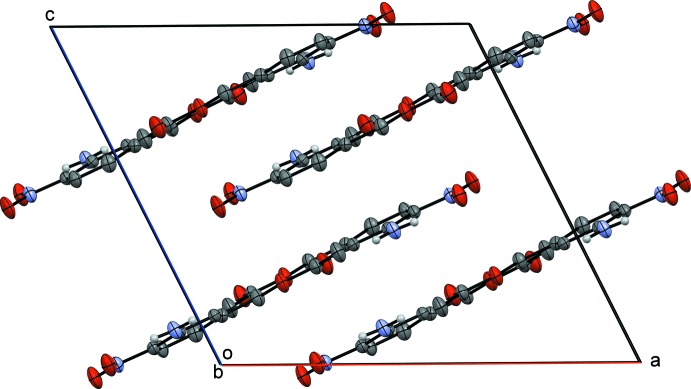
A displacement ellipsoid plot of the unit cell of 3-amino-4-nitro­benzoic acid observed down the *b* axis.

The crystal structure of 3-amino-4-nitro­benzyl acetate has inter­esting supra­molecular features. The mol­ecules are arranged in layers held together by inter­molecular N2—H2*A*⋯O4 [3.005 (2) Å] hydrogen bonding [graph set *C*1,1(9)] inter­actions between the carbonyl and amine groups forming a zigzag chain along the *b*-axis direction (Fig. 2 ▸ and Table 1 ▸) lying in a plane parallel to (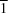
02). A view of a single layer along the *ab* plane, observed down the *c* axis (Fig. 2 ▸) provides a representative illustration of the hydrogen-bonding inter­actions of 3-amino-4-nitro­benzyl acetate. Observing the unit cell along the *b*-axis (Fig. 3 ▸) shows four layers along the *c* axis separated at a distance of 3.3163 (10) Å with the arene groups stacked one above the other. The chains stack along the *c* axis by π–π inter­actions [centroid–centroid distances = 3.6240 (3) Å (symmetry code 1 − *x*, 1 − *y*, 1 − *z*) and 3.5855 (4) Å (symmetry code 1 − *x*, *y*, 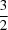 − *z*)].

## Database Survey   

For a related benzyl acetate structure, see Kasuga *et al.* (2015 ▸). For alkyl- and aryl-3-amino-4-nitro-benzoates and benzoic acids displaying similar intramolecular hydrogen bonding between the amino and nitro groups, see: Narendra Babu *et al.* (2009 ▸); Abdul Rahim *et al.* (2010 ▸); Yoon *et al.* (2011 ▸); Yoon *et al.* (2012 ▸). 

## Synthesis and Crystallization   

**(5-Amino-2-nitro­phen­yl)methanol:** (5-amino-2-nitro­phen­yl)methanol was prepared by a modified literature protocol (Yoon *et al.* 1973 ▸). To a solution of 5-amino-2-nitro­benzoic acid (97%, 1.5 g, 8.2 mmol) dissolved in tetra­hydro­furan (10 mL), borane–THF (27.6 mL, 1.0 *M* in THF, 27.6 mmol) was added dropwise by dropping funnel over 30 minutes. The reaction was stirred overnight at room temperature. The reaction was quenched with aqueous potassium hydroxide (2.45 *M*) until pH 11 was reached and continued to be stirred for 6 h, resulting in a greenish-brown solution. The solution was treated with a saturated solution of potassium carbonate followed by treatment with hydro­chloric acid until pH 1 was reached. The reaction mixture was extracted with diethyl ether three times; organic portions were collected and dried with anhydrous sodium sulfate overnight. The solution was filtered under vacuum, the filtrate was collected and all solvent removed under rotary evaporation to give a green powder (0.68 g, 49%). ^1^H NMR, (300 MHz, acetone-*d*_6_) δ: 4.61 (*t*, 1H, –OH, ^3^ *J*_HH_ = 5.3 Hz), 4.95 (*d*, 2H, CH_2_, ^3^ *J*_HH_ = 5.3 Hz) , 6.03 (*bs*, 2H, NH_2_), 6.63 (*d*d, 1H, Ar-H, ^3^ *J*_HH_ = 8.8 Hz, ^3^ *J*_HH_ = 2.3 Hz), 7.07 (*m*, 1H, Ar-H), 8.02 (*dd*, 1H, ^3^ *J*_HH_ = 9.4 Hz, ^3^ *J*_HH_ = 3.0 Hz) (Aujard *et al.* 2006 ▸). Note: minor impurities were observed in the base line in the aromatic region.

**3-Amino-4-nitro­benzyl acetate**: (5-amino-2-nitro­phen­yl)methanol (10 mg, 0.0595 mmol) and tri­ethyl­amine (17 µL, 0.119 mmol) were dissolved in aceto­nitrile-*d*_6_ (0.7 mL) and added to an NMR tube. Acetic anhydride (11.2 µL, 0.119 mmol) was added to the tube *via* a syringe. The tube was held at room temperature overnight. On completion of the reaction the solvent was removed *in vacuo* and the residue was reconstituted in a minimum amount of methyl­ene chloride. The sample was loaded on a column of silica and eluted with an ethyl acetate/hexane solution (70/30 *v*/*v* %). The separated solutions were allowed to slowly evaporate at room temperature. The parent compound (5-amino-2-nitro­benzyl acetate) elutes first and is isolated as a yellow powder. ^1^H NMR (300 MHz, CDCl_3_) δ: 2.10 (*s*, 3H, C*H*_3_), 4.35 (*bs*, 2H, N*H*_2_), 5.50 (*s*, 2H, C*H*_2_), 6.55 (*dd*, 1H, Ar-H, **^3^** *J* **_HH_** = 8.9 Hz, **^5^** *J* **_HH_** = 2.5 Hz), 6.68 (*m*, 1H, Ar-H), 8.09 (*dd*, 1H, Ar-H, **^3^** *J* **_HH_** = 8.9 Hz, **^5^** *J* **_HH_** = 2.5 Hz) (Serafinowski *et al.* 2008 ▸). Yellow crystals of the title compound were isolated (less than 1 mg) in later eluate. ^1^H NMR (300 MHz, CDCl_3_) δ: 2.19 (*s*, 3H, C*H*_3_), 5.53 (*s*, 2H, C*H*_2_), 7.44 (*bs*, 2H, N*H*_2_), 7.65 (*dd*, 1H, Ar-H, **^3^** *J* **_HH_** = 8.9 Hz, **^5^** *J* **_HH_** = 2.5 Hz), 7.75 (m, 1H, Ar-H), 8.15 (*d*, 1H, Ar-H, **^3^** *J* **_HH_** = 8.9 Hz).

### Refinement   

Crystal data, data collection and structure refinement details are summarized in Table 2 ▸. Hydrogen atoms were refined freely.

## Supplementary Material

Crystal structure: contains datablock(s) I. DOI: 10.1107/S2056989015008750/pk2548sup1.cif

Structure factors: contains datablock(s) I. DOI: 10.1107/S2056989015008750/pk2548Isup2.hkl

Supporting information file. DOI: 10.1107/S2056989015008750/pk2548Isup3.cml

CCDC reference: 1063364

Additional supporting information:  crystallographic information; 3D view; checkCIF report

## Figures and Tables

**Table 1:** Hydrogen-bond geometry (, )

*D*H*A*	*D*H	H*A*	*D* *A*	*D*H*A*
N2H2*A*O4^i^	0.83(2)	2.18(2)	3.005(2)	171.5(17)
N2H2*B*O1	0.84(2)	2.06(2)	2.6600(19)	128.0(16)
N2H2*B*O1^ii^	0.84(2)	2.44(2)	3.1443(19)	142.7(16)

Symmetry codes: (i) 
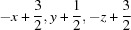
; (ii) 
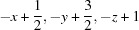
.

**Table 2:** Experimental details

Crystal data	
Chemical formula	C_9_H_10_N_2_O_4_
*M*_r_	210.19
Crystal system, space group	Monoclinic, *C*2/*c*
Temperature (K)	173
*a*, *b*, *c* ()	14.4803(15), 11.4054(11), 13.0936(13)
()	116.341(8)
*V* (^3^)	1937.9(4)
*Z*	8
Radiation type	Mo *K*
(mm^1^)	0.12
Crystal size (mm)	0.25 0.25 0.10

Data collection	
Diffractometer	Rigaku Mercury375R
Absorption correction	Multi-scan (*REQAB*; Rigaku, 1998 ▸)
*T*_min_, *T*_max_	0.840, 1.000
No. of measured, independent and observed [*I* > 2(*I*)] reflections	8409, 1759, 1348
*R*_int_	0.045
(sin /)_max_ (^1^)	0.601

Refinement	
*R*[*F* ^2^ > 2(*F* ^2^)], *wR*(*F* ^2^), *S*	0.037, 0.098, 1.06
No. of reflections	1759
No. of parameters	176
H-atom treatment	All H-atom parameters refined
_max_, _min_ (e ^3^)	0.21, 0.17

Computer programs: *CrystalClear-SM Expert* (Rigaku, 2014), *SHELXT* (Sheldrick, 2015*a* ▸), *SHELXL2013* (Sheldrick, 2015*b* ▸) and *OLEX2* (Dolomanov *et al.*, 2009 ▸).

## supplementary crystallographic information

### Crystal data

**Table:**

C_9_H_10_N_2_O_4_	*F*(000) = 880
*M**_r_* = 210.19	*D*_x_ = 1.441 Mg m^−^^3^
Monoclinic, *C*2/*c*	Mo *K*α radiation, λ = 0.71075 Å
*a* = 14.4803 (15) Å	Cell parameters from 513 reflections
*b* = 11.4054 (11) Å	θ = 1.6–25.4°
*c* = 13.0936 (13) Å	µ = 0.12 mm^−^^1^
β = 116.341 (8)°	*T* = 173 K
*V* = 1937.9 (4) Å^3^	Prism, yellow
*Z* = 8	0.25 × 0.25 × 0.10 mm

### Data collection

**Table:**

Rigaku Mercury375R (2x2 bin mode) diffractometer	1759 independent reflections
Radiation source: Sealed Tube	1348 reflections with *I* > 2σ(*I*)
Graphite Monochromator monochromator	*R*_int_ = 0.045
Detector resolution: 13.6612 pixels mm^-1^	θ_max_ = 25.3°, θ_min_ = 2.4°
profile data from ω scans	*h* = −17→17
Absorption correction: multi-scan (*REQAB*; Rigaku, 1998)	*k* = −13→13
*T*_min_ = 0.840, *T*_max_ = 1.000	*l* = −15→15
8409 measured reflections	

### Refinement

**Table:**

Refinement on *F*^2^	Primary atom site location: structure-invariant direct methods
Least-squares matrix: full	Hydrogen site location: difference Fourier map
*R*[*F*^2^ > 2σ(*F*^2^)] = 0.037	All H-atom parameters refined
*wR*(*F*^2^) = 0.098	*w* = 1/[σ^2^(*F*_o_^2^) + (0.0578*P*)^2^ + 0.2118*P*] where *P* = (*F*_o_^2^ + 2*F*_c_^2^)/3
*S* = 1.06	(Δ/σ)_max_ < 0.001
1759 reflections	Δρ_max_ = 0.21 e Å^−^^3^
176 parameters	Δρ_min_ = −0.17 e Å^−^^3^
0 restraints	

### Special details

**Table:**

Geometry. All e.s.d.'s (except the e.s.d. in the dihedral angle between two l.s. planes) are estimated using the full covariance matrix. The cell e.s.d.'s are taken into account individually in the estimation of e.s.d.'s in distances, angles and torsion angles; correlations between e.s.d.'s in cell parameters are only used when they are defined by crystal symmetry. An approximate (isotropic) treatment of cell e.s.d.'s is used for estimating e.s.d.'s involving l.s. planes.

### Fractional atomic coordinates and isotropic or equivalent isotropic displacement parameters (Å^2^)

**Table:**

	*x*	*y*	*z*	*U*_iso_*/*U*_eq_	
O3	0.74138 (8)	0.43515 (9)	0.74195 (10)	0.0324 (3)	
O1	0.23008 (8)	0.61948 (10)	0.50492 (11)	0.0418 (3)	
O2	0.18245 (8)	0.43814 (10)	0.46692 (11)	0.0436 (3)	
O4	0.86707 (9)	0.30050 (11)	0.79735 (12)	0.0456 (4)	
N1	0.25141 (9)	0.51321 (11)	0.50551 (11)	0.0288 (3)	
N2	0.42892 (12)	0.67596 (12)	0.60612 (12)	0.0300 (3)	
H2A	0.4821 (16)	0.7166 (16)	0.6337 (16)	0.036 (5)*	
H2B	0.3700 (16)	0.7046 (15)	0.5821 (16)	0.039 (5)*	
C6	0.43920 (11)	0.55950 (12)	0.60073 (12)	0.0228 (3)	
C1	0.35704 (10)	0.47782 (13)	0.55271 (12)	0.0245 (3)	
C4	0.55919 (11)	0.39372 (13)	0.64720 (12)	0.0252 (3)	
C5	0.54112 (10)	0.51152 (13)	0.64747 (11)	0.0228 (3)	
H5	0.5978 (13)	0.5675 (14)	0.6814 (13)	0.024 (4)*	
C2	0.37680 (12)	0.35678 (14)	0.54982 (13)	0.0295 (4)	
H2	0.3212 (13)	0.3041 (14)	0.5154 (15)	0.031 (4)*	
C3	0.47512 (12)	0.31496 (14)	0.59587 (14)	0.0312 (4)	
H3	0.4895 (13)	0.2318 (16)	0.5930 (15)	0.033 (4)*	
C7	0.66619 (11)	0.34199 (14)	0.69873 (14)	0.0296 (4)	
H7A	0.6780 (14)	0.2874 (16)	0.7620 (16)	0.040 (5)*	
H7B	0.6772 (12)	0.2957 (14)	0.6407 (15)	0.032 (4)*	
C8	0.84105 (11)	0.40198 (15)	0.79024 (13)	0.0307 (4)	
C9	0.91100 (13)	0.50537 (18)	0.83137 (18)	0.0419 (5)	
H9A	0.8961 (16)	0.5562 (19)	0.7680 (19)	0.055 (6)*	
H9B	0.9809 (17)	0.4806 (16)	0.8703 (17)	0.046 (5)*	
H9C	0.8934 (17)	0.554 (2)	0.881 (2)	0.067 (7)*	

### Atomic displacement parameters (Å^2^)

**Table:**

	*U*^11^	*U*^22^	*U*^33^	*U*^12^	*U*^13^	*U*^23^
O3	0.0175 (5)	0.0288 (6)	0.0445 (7)	0.0021 (4)	0.0080 (5)	−0.0018 (5)
O1	0.0242 (6)	0.0319 (7)	0.0621 (8)	0.0054 (5)	0.0128 (6)	0.0060 (5)
O2	0.0194 (6)	0.0411 (7)	0.0601 (8)	−0.0073 (5)	0.0085 (6)	−0.0033 (6)
O4	0.0271 (6)	0.0400 (8)	0.0605 (8)	0.0089 (5)	0.0111 (6)	−0.0009 (6)
N1	0.0188 (6)	0.0314 (8)	0.0327 (7)	0.0002 (5)	0.0083 (5)	0.0031 (6)
N2	0.0199 (7)	0.0271 (8)	0.0379 (8)	0.0002 (6)	0.0082 (6)	−0.0025 (6)
C6	0.0214 (7)	0.0266 (8)	0.0210 (7)	0.0013 (6)	0.0098 (6)	0.0011 (6)
C1	0.0180 (7)	0.0307 (8)	0.0234 (7)	0.0004 (6)	0.0078 (6)	0.0027 (6)
C4	0.0210 (7)	0.0312 (8)	0.0239 (7)	0.0010 (6)	0.0104 (6)	0.0002 (6)
C5	0.0198 (8)	0.0271 (8)	0.0212 (7)	−0.0025 (6)	0.0086 (6)	−0.0011 (6)
C2	0.0218 (8)	0.0283 (9)	0.0358 (9)	−0.0063 (7)	0.0105 (7)	−0.0029 (7)
C3	0.0279 (8)	0.0238 (9)	0.0406 (9)	−0.0004 (6)	0.0142 (7)	−0.0017 (7)
C7	0.0237 (8)	0.0262 (8)	0.0363 (9)	0.0007 (6)	0.0110 (7)	−0.0024 (7)
C8	0.0211 (8)	0.0379 (10)	0.0305 (8)	0.0056 (7)	0.0091 (7)	0.0011 (7)
C9	0.0214 (9)	0.0483 (12)	0.0489 (11)	−0.0017 (8)	0.0091 (8)	−0.0015 (9)

### Geometric parameters (Å, º)

**Table:**

O3—C7	1.4449 (19)	C4—C3	1.419 (2)
O3—C8	1.3477 (19)	C4—C7	1.509 (2)
O1—N1	1.2500 (16)	C5—H5	0.978 (17)
O2—N1	1.2401 (17)	C2—H2	0.944 (17)
O4—C8	1.208 (2)	C2—C3	1.362 (2)
N1—C1	1.4303 (19)	C3—H3	0.975 (18)
N2—H2A	0.83 (2)	C7—H7A	0.988 (19)
N2—H2B	0.83 (2)	C7—H7B	0.994 (17)
N2—C6	1.342 (2)	C8—C9	1.491 (3)
C6—C1	1.419 (2)	C9—H9A	0.96 (2)
C6—C5	1.4320 (19)	C9—H9B	0.95 (2)
C1—C2	1.414 (2)	C9—H9C	0.97 (2)
C4—C5	1.369 (2)		
			
C8—O3—C7	116.19 (12)	C3—C2—C1	120.94 (14)
O1—N1—C1	119.36 (12)	C3—C2—H2	119.5 (10)
O2—N1—O1	121.00 (12)	C4—C3—H3	118.7 (10)
O2—N1—C1	119.64 (13)	C2—C3—C4	119.75 (15)
H2A—N2—H2B	122.7 (17)	C2—C3—H3	121.5 (10)
C6—N2—H2A	118.1 (12)	O3—C7—C4	109.47 (13)
C6—N2—H2B	119.1 (12)	O3—C7—H7A	108.3 (11)
N2—C6—C1	125.60 (13)	O3—C7—H7B	109.9 (9)
N2—C6—C5	118.24 (13)	C4—C7—H7A	112.5 (10)
C1—C6—C5	116.16 (13)	C4—C7—H7B	110.3 (10)
C6—C1—N1	122.12 (13)	H7A—C7—H7B	106.2 (14)
C2—C1—N1	117.02 (13)	O3—C8—C9	111.22 (14)
C2—C1—C6	120.86 (13)	O4—C8—O3	122.52 (15)
C5—C4—C3	119.84 (14)	O4—C8—C9	126.26 (15)
C5—C4—C7	122.83 (14)	C8—C9—H9A	108.1 (13)
C3—C4—C7	117.33 (14)	C8—C9—H9B	110.5 (11)
C6—C5—H5	116.3 (9)	C8—C9—H9C	110.8 (13)
C4—C5—C6	122.40 (13)	H9A—C9—H9B	115.0 (17)
C4—C5—H5	121.3 (9)	H9A—C9—H9C	102.2 (18)
C1—C2—H2	119.6 (10)	H9B—C9—H9C	110.0 (17)
			
O1—N1—C1—C6	0.9 (2)	C5—C6—C1—N1	178.48 (13)
O1—N1—C1—C2	−179.17 (13)	C5—C6—C1—C2	−1.44 (19)
O2—N1—C1—C6	−178.35 (13)	C5—C4—C3—C2	−1.7 (2)
O2—N1—C1—C2	1.6 (2)	C5—C4—C7—O3	−2.0 (2)
N1—C1—C2—C3	−178.07 (14)	C3—C4—C5—C6	2.1 (2)
N2—C6—C1—N1	−1.2 (2)	C3—C4—C7—O3	177.40 (13)
N2—C6—C1—C2	178.91 (14)	C7—O3—C8—O4	0.1 (2)
N2—C6—C5—C4	179.13 (13)	C7—O3—C8—C9	179.98 (14)
C6—C1—C2—C3	1.9 (2)	C7—C4—C5—C6	−178.49 (13)
C1—C6—C5—C4	−0.5 (2)	C7—C4—C3—C2	178.86 (15)
C1—C2—C3—C4	−0.2 (2)	C8—O3—C7—C4	−179.68 (12)

### Hydrogen-bond geometry (Å, º)

**Table:**

*D*—H···*A*	*D*—H	H···*A*	*D*···*A*	*D*—H···*A*
N2—H2*A*···O4^i^	0.83 (2)	2.18 (2)	3.005 (2)	171.5 (17)
N2—H2*B*···O1	0.84 (2)	2.06 (2)	2.6600 (19)	128.0 (16)
N2—H2*B*···O1^ii^	0.84 (2)	2.44 (2)	3.1443 (19)	142.7 (16)

Symmetry codes: (i) −*x*+3/2, *y*+1/2, −*z*+3/2; (ii) −*x*+1/2, −*y*+3/2, −*z*+1.
